# Vaccine communication: a critical review of foundations, challenges, and future directions

**DOI:** 10.1038/s41541-026-01487-9

**Published:** 2026-05-18

**Authors:** Xiaoli Nan

**Affiliations:** University of Maryland, College Park, USA

**Keywords:** Cultural and media studies, Cultural and media studies, Politics and international relations, Science, technology and society, Social policy

## Abstract

Vaccination remains one of the most effective public health interventions, yet sustaining uptake is increasingly challenged by complex informational and political environments. This review provides a targeted synthesis of existing evidence on vaccine communication, examining its foundations, emerging challenges, and future directions. It underscores the need for strategies that address misinformation, polarization, evolving policy contexts, and declining trust, while also considering emerging tools that may enhance communication and strengthen the resilience of immunization systems.

Vaccination remains one of the most effective public health interventions for preventing infectious diseases and reducing global morbidity and mortality. Yet achieving and sustaining high vaccine uptake is never guaranteed. Communication—the transmission of information, ideas, and values through symbolic processes^1^—plays a central role in shaping vaccine confidence, addressing hesitancy, and guiding public health behavior. Successful immunization campaigns, such as those targeting smallpox and polio, demonstrate how clear messaging, trusted institutions, and coordinated public health infrastructures can translate scientific advances into widespread population protection.

Importantly, however, these successes were not driven by communication alone. The high transmissibility, visible symptoms, and severe consequences of these diseases created strong, shared perceptions of risk, which likely enhanced public receptivity to vaccination messages. In this sense, the diseases themselves functioned as powerful “signals” that reinforced the urgency and credibility of public health communication. By contrast, more recent vaccination efforts—most notably during the COVID-19 pandemic—have unfolded under conditions in which perceived risk has been more variable and, at times, contested. These differences highlight that the effectiveness of vaccine communication is shaped not only by message design and institutional trust but also by how populations perceive the threat posed by the disease itself.

Additionally, the communication environment surrounding vaccines has become increasingly complex. The COVID-19 pandemic highlighted both the importance and the fragility of vaccine communication. Rapid scientific progress enabled the development of new vaccines in record time, yet the rollout occurred in a highly contested informational landscape. Digital media ecosystems accelerated the spread of misinformation and disinformation, political polarization shaped interpretations of public health guidance, and evolving scientific evidence created uncertainty that was often exploited by competing narratives. These dynamics contributed to declining trust in public health institutions in some contexts and revealed the limitations of traditional communication approaches that rely primarily on one-way dissemination of scientific information.

Against this backdrop, this review provides a targeted synthesis of existing evidence on vaccine communication, examining its foundations, emerging challenges, and future directions. The synthesis is targeted in that it is a focused, theory-informed integration of key findings from prior research, prioritizing studies and frameworks most relevant to contemporary challenges rather than attempting an exhaustive or fully systematic review.

The review first defines key concepts and frameworks that shape research on vaccine hesitancy and vaccine decision-making. It then reviews evidence-based communication strategies developed over several decades of research. The review next examines emerging challenges confronting vaccine communication, including the spread of misinformation and disinformation, increasing political polarization, shifting vaccine policy debates, and declining institutional trust. In particular, it interrogates communication strategies that can be deployed to address these evolving challenges. Finally, it highlights emerging frontiers and innovative tools—ranging from social listening systems and artificial intelligence to behavioral nudges and multi-stakeholder partnerships—that may help strengthen vaccine communication in rapidly evolving information ecosystems. By integrating insights from past research with lessons from the COVID-19 era, this review aims to inform future communication strategies that enhance vaccine confidence, support immunization uptake, and strengthen the resilience of public health systems—that is, their capacity to maintain and restore essential immunization functions in the face of disruptions such as disease outbreaks, misinformation, policy changes, or declining public trust. In this context, effective communication contributes to resilience by sustaining public confidence, promoting timely vaccine uptake, and preventing localized disruptions from cascading into broader declines in coverage.

## Defining Vaccine Communication and Vaccine Hesitancy

### Scope and goals of vaccine communication

Vaccine communication, like all forms of health communication, operates across multiple, interconnected levels^2^. At the intrapersonal level, it is embedded in individual cognition, emotions, and risk perceptions, which determine vaccine acceptance. At the interpersonal level, it occurs in direct conversations between providers and patients or among peers. At the group level, vaccine communication unfolds within families, peer networks, and community associations, where collective norms strongly influence individual choices. At the organizational level, conversations at health systems, schools, and workplaces play a role in shaping policies and practices that structure vaccine decision-making. Finally, at the societal level, it takes place in mass media, government campaigns, and broader cultural narratives, which create the backdrop against which individual and collective vaccination decisions are made. Collectively, these levels underscore the fact that vaccine communication is not confined to clinical encounters but permeates the wider social fabric, shaping how individuals and communities understand and act on vaccination.

At its core, vaccine communication is the process through which facts, values, social norms, and perceptions of benefits and risks are exchanged among health authorities, providers, communities, and individuals. Its goals are multifaceted. First, vaccine communication seeks to inform—delivering accurate, timely, and accessible information about the safety, efficacy, and availability of vaccines, as well as the risks posed by vaccine-preventable diseases. Second, it aims to motivate action by addressing hesitancy, reinforcing social responsibility, and promoting confidence in vaccination as both an individual choice and a collective good. Equally important, vaccine communication works to build and sustain trust—trust in the science of vaccination, in the systems that deliver it, and in the institutions that recommend it.

### Communication as part of immunization system resilience

Effective vaccine communication is integral to the resilience of immunization programs. Beyond conveying essential information about vaccine schedules and access, strong communication strategies help build public confidence in new and emerging vaccines, including those introduced under an Emergency Use Authorization (EUA) during a pandemic. They also support the sustained uptake of routine childhood vaccines such as measles, mumps, and rubella (MMR) and contribute to improving coverage for newer vaccines such as the human papillomavirus (HPV) vaccine. Communication further functions as a stabilizing force during supply disruptions, policy changes, or safety concerns, reducing the likelihood that isolated challenges escalate into widespread distrust or declining coverage.

Resilience in immunization programs depends on embedding communication across every stage—planning, rollout, monitoring, and crisis response—so that communities remain informed, engaged, and prepared. When communication is treated as a core pillar rather than an ancillary function, immunization systems are better equipped to anticipate challenges, adapt to changing conditions, and sustain long-term vaccine confidence. Increasingly, effective communication is also required to counter vaccine misinformation, which has been amplified by new technologies and deepening institutional distrust. The paradox of using credible, evidence-based communication to counter misleading or manipulative narratives reflects the realities of an era shaped by digital media, rapid information flows, and political polarization.

### Defining vaccine hesitancy

A central aim of vaccine communication is to foster public confidence in vaccines and reduce vaccine hesitancy. The Strategic Advisory Group of Experts (SAGE) on Immunization defines vaccine hesitancy as the “delay in acceptance or refusal of vaccines despite the availability of vaccination services”^3^. Hesitancy is widely recognized as complex, context-specific, and influenced by a range of factors that vary across time, place, and specific vaccines^4^.

Importantly, vaccine hesitancy is not synonymous with outright refusal or organized anti-vaccine activism. Many individuals who express hesitancy ultimately choose to vaccinate, but may do so with delay, uncertainty, or selective acceptance^5^. Determinants of hesitancy span several levels: individual factors such as knowledge, beliefs, and risk perceptions; social and cultural influences including peer norms, religious values, and political identity; structural barriers such as cost, transportation, or inconvenient clinic hours; and historical and systemic issues such as medical racism and long-standing mistrust of government institutions^5,6^.

### Determinants of vaccine hesitancy

Understanding why individuals delay or refuse vaccination is central to effective communication and program design. The World Health Organization’s SAGE introduced the 3Cs framework—Confidence, Complacency, and Convenience—and a broader Determinants Matrix that considers contextual, individual/group, and vaccine-specific influences^3^. Building on this, the 5Cs model adds Calculation, which captures deliberative risk–benefit weighing, and Collective Responsibility, reflecting prosocial motivation to protect others. The 5Cs scale has been validated in multiple settings and remains a widely used tool for monitoring psychological antecedents of vaccination^4^.

Confidence—trust in the safety and effectiveness of vaccines, healthcare systems, and vaccine policy makers—consistently emerges as a high-leverage determinant of vaccine hesitancy. A systematic review of global vaccine confidence found substantial cross-national variability: confidence in vaccine safety and effectiveness was relatively high in many South Asian and sub-Saharan African countries but markedly lower in parts of Europe, where skepticism about safety was more prevalent^7^. Low confidence in vaccine safety is among the strongest predictors of hesitancy across different populations and vaccine types^5,6^. Evidence from COVID-19 research reinforces this point: rapid reviews and meta-analyses consistently show that concerns about vaccine safety, speed of development, and distrust in institutions are primary drivers of hesitancy^8,9^.

Complacency, or the perception that vaccine-preventable diseases pose little or no risk, is another influential determinant of vaccine hesitancy. Rapid reviews conducted during the COVID-19 pandemic found that lower perceived susceptibility and severity were strongly associated with vaccine hesitancy, whereas higher perceived risk reduced hesitancy across populations^10^. Similar patterns have been documented across other vaccines: a systematic review of parental attitudes toward childhood immunization identified low perceived risk of disease as a recurrent reason for hesitancy^5^, while a meta-analysis of H1N1 vaccination during the 2009 influenza pandemic showed that higher perceived susceptibility and severity significantly predicted uptake^11^.

The Constraints or Convenience dimension highlights structural barriers—such as transportation, clinic hours, childcare, cost, and documentation—that can impede vaccination even when attitudes are favorable. SAGE’s report emphasized that in some contexts, service deficits rather than attitudinal hesitancy are the primary barrier to uptake^3^. A systematic review further demonstrates that reducing these frictions—through reminders, defaults, and on-site availability—can significantly increase coverage^12^.

Calculation refers to individuals’ engagement in extensive information searching, a process particularly vulnerable to misinformation and uncertainty. Research indicates that higher levels of calculation are often linked to greater vaccine hesitancy rather than increased uptake^4^. Evidence from the COVID-19 pandemic supports this pattern: individuals with stronger information-seeking tendencies were found to be more susceptible to misinformation and conspiracy beliefs, both of which predicted higher levels of hesitancy across diverse countries^8,9^.

Collective Responsibility—the willingness to vaccinate to protect others—has consistently emerged as a strong predictor of vaccine acceptance. This dimension captures prosocial motives, such as protecting vulnerable community members, and is reinforced by perceptions of social norms. Individuals who endorse prosocial values or perceive higher levels of community vaccination are more likely to accept vaccines^13^. A meta-analysis of influenza vaccination found that altruistic motives, such as protecting children or the elderly, reliably predicted uptake across diverse populations^14^.

In addition to the 5Cs, interpersonal dynamics also matter. Research on HPV vaccine uptake demonstrated that strong, clear provider recommendations using presumptive language significantly improved vaccine acceptance^15^. Additionally, sociodemographic, cultural, and political factors—such as younger age, lower income or education, minority status, and political ideology—are associated with hesitancy, although these effects are context-specific and often reflect broader structural and informational inequities^16^. Determinants can also vary by vaccine type. For instance, COVID-19 vaccine hesitancy has been shaped by unique factors such as the rapid development of mRNA technologies and shifting public health guidance^17^.

Taken together, these findings illustrate that the determinants of vaccine hesitancy are multidimensional and context dependent, often reflecting a complex interplay among trust, risk appraisal, structural access, deliberative processing, and prosocial norms. For practitioners and policymakers, this means that effective communication strategies should be embedded within broader efforts to build confidence and improve access, using diagnostic tools such as the 3Cs/5Cs models^3,4^ to guide tailored interventions.

## Evidence-Based Communication Strategies

Decades of research show that vaccine communication, when tailored to specific audiences and delivered through trusted sources, can strengthen confidence and reduce hesitancy. Findings from meta-analyses and systematic reviews identify a range of evidence-based approaches that continue to shape and inform best practices in the field.

### Message framing

The way vaccine benefits and risks are presented can shape perceptions, though the effects are often modest. In a meta-analysis of 32 studies, O’Keefe and Nan^18^ found no overall persuasive advantage of gain-framed messages (emphasizing benefits) over loss-framed messages (emphasizing costs of non-action) for vaccination. However, certain subgroups—such as parents making decisions for children—showed slightly stronger responses to loss frames. A qualitative synthesis suggests that effects of message framing in vaccine communication often depend on characteristics of the message recipient, perceived risk, or situational factors such as disease prevalence and vaccine novelty^19^.

### Narrative and storytelling

Narratives—such as personal stories or testimonials—can make health risks more concrete and emotionally salient, thereby enhancing message persuasiveness. A meta-analysis on narrative persuasion in health communication found that narratives generally had a small overall effect, but those delivered through audio or video significantly influenced attitudes and behaviors, whereas print-based narratives showed no significant impact^20^. The effectiveness of narratives also appears to vary by health topic. Messages advocating detection and prevention behaviors demonstrated stronger effects, while those promoting cessation behaviors did not yield significant changes^20^. Experimental evidence further highlights the value of combining narrative and statistical information: hybrid messages containing both narrative and statistical descriptions of HPV risks led to higher perceived susceptibility than either format alone^21^. Additionally, first-person narratives were more effective than third-person accounts in elevating risk perceptions about HPV, underscoring the importance of message perspective in shaping audience responses^21^.

### Trusted messengers

Source credibility, which encompasses both trustworthiness and expertise, is a well-documented driver of message persuasiveness. Likewise, messenger trustworthiness and expertise play a central role in vaccine acceptance. Systematic reviews consistently show that healthcare providers are among the most trusted sources of vaccine information, and that strong, clear recommendations from providers significantly increase uptake across childhood, adolescent, and adult vaccines^5,22^. During the COVID-19 pandemic, trust in scientists and health professionals was strongly associated with vaccine acceptance, whereas reliance on information from social media influencers or politically motivated actors was linked to greater hesitancy^8,23^.

### Community engagement and participatory design

Engaging communities in the development of communication materials fosters cultural relevance and trust. Systematic reviews^24^ note that co-designing interventions with target populations—using focus groups or advisory boards—can improve acceptability and, in some cases, vaccination rates. These approaches move beyond one-way information provision toward partnership, which is particularly important in historically marginalized communities.

### Policy communication

Policy tools such as mandates and incentives require careful, transparent communication. Petersen et al.^25^ emphasize that disclosure of both positive and negative vaccine information is critical for maintaining public trust. Evidence shows that while transparent communication about potential risks may temporarily reduce vaccine acceptance, it ultimately strengthens trust in health authorities. By contrast, vague or overly reassuring messaging does not improve acceptance and may instead erode trust while fueling conspiracy beliefs.

### Conclusion

Taken together, these findings indicate that effective vaccine communication should combine multiple elements: thoughtful framing and narrative, trusted messengers, community participation, and transparent policy communication. No single approach suffices across all contexts, but integrating these strategies increases the likelihood of improving vaccine confidence and uptake.

However, despite empirical support for these strategies, their effectiveness is neither universal nor guaranteed. The COVID-19 pandemic, in particular, revealed the limits of traditional vaccine communication approaches when confronted with pervasive mis- and disinformation and deepening political polarization. In highly fragmented information environments, vaccine communication may compete with emotionally resonant falsehoods that spread more quickly and widely^26^. Moreover, when vaccine attitudes are intertwined with political identity or broader distrust in government, science, and media institutions, even well-designed communication delivered by credible experts may have limited persuasive impact. More recent vaccine policy changes under the Trump administration, including actions led by Health and Human Services Secretary Robert F. Kennedy Jr. that have altered aspects of federal vaccine oversight, advisory processes, and regulatory frameworks, have further intensified debate around the role of government, scientific authority, and parental autonomy in vaccination policy, potentially fueling vaccine hesitancy. These developments underscore that communication does not operate in a vacuum: its effects are conditioned by broader social, political, and institutional dynamics. Recognizing these constraints is essential for understanding why traditional message strategies sometimes fall short and why contemporary vaccine communication must grapple with structural and contextual challenges beyond message design alone.

## Emerging Challenges in Vaccine Communication

**Fig. 1:**
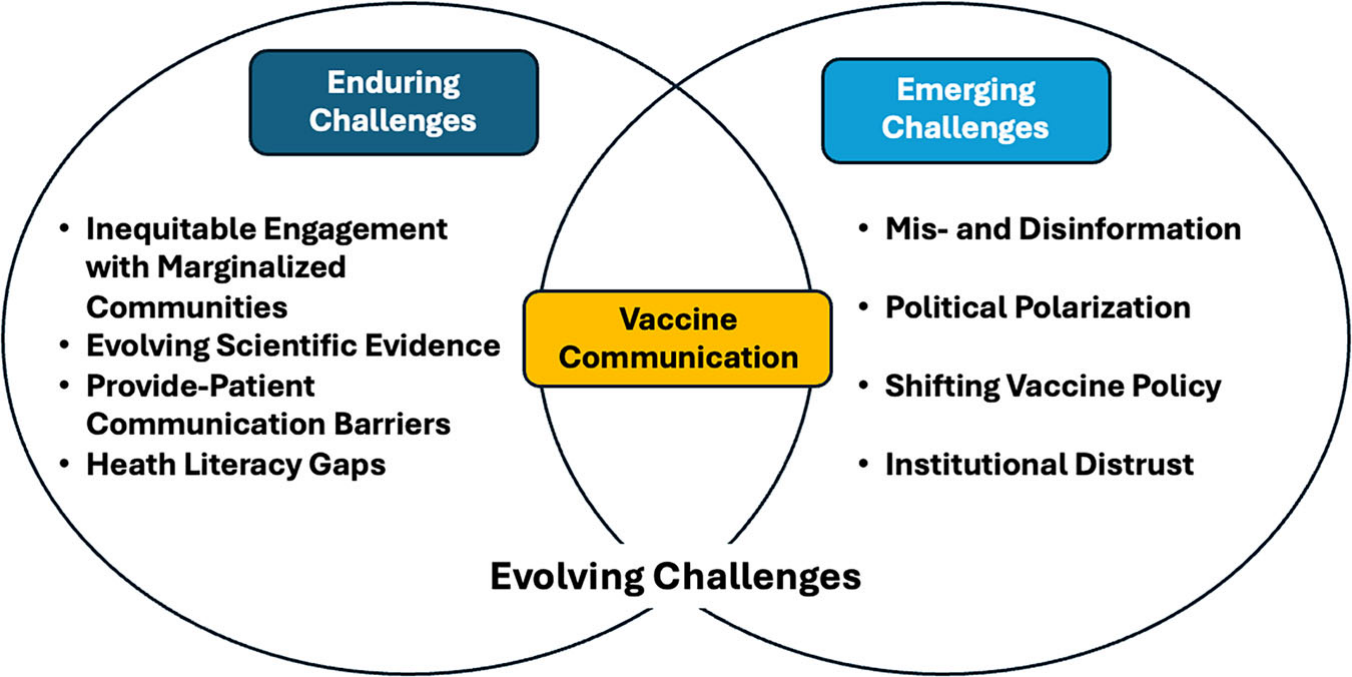
The Evolving Challenges of Vaccine Communication.

While vaccine communication has long faced enduring challenges—including inequitable engagement with marginalized communities^27^, evolving scientific evidence^28,29^, provider–patient communication barriers^30^, and health literacy gaps^31,32^—the most pressing challenges today are structural and political. In the aftermath of COVID-19, vaccine communication in the U.S. and some other parts of the world increasingly occurs within environments shaped by pervasive misinformation and disinformation, deepening political polarization, and significant shifts in vaccine policy (see Fig. 1). Together with partisan media ecosystems, these developments have contributed to heightened distrust of government and public health institutions, substantially complicating the task of sustaining vaccine confidence and uptake.

### Misinformation and disinformation

Vaccine communication now unfolds within information ecosystems where falsehoods spread rapidly, strategically, and at scale. The COVID-19 pandemic did not create vaccine misinformation, but it dramatically intensified its volume and visibility. Even brief exposure to online vaccine misinformation has been shown to measurably depress vaccination intent^33^. Systematic reviews document the scale of the problem across platforms, the centrality of vaccines as a recurring misinformation topic, and the role of algorithmic amplification and engagement-driven platform architectures in privileging emotionally arousing or controversial content^34,35^.

During the pandemic, misinformation narratives evolved rapidly—from claims about microchips and infertility to doubts about mRNA technology, exaggerated accounts of adverse events, and conspiracy theories linking vaccines to broader agendas. The speed of scientific updates, shifting public health guidance (e.g., changing mask and booster recommendations), and the novelty of mRNA platforms created informational ambiguity that misinformation actors exploited. In this context, the challenge was not simply correcting discrete false claims but responding to a continuously adapting ecosystem of narratives that blended partial truths and uncertainty.

Beyond organic virality, coordinated influence operations have deliberately weaponized the vaccine debate. Analyses of Twitter/X identified Russian state-linked troll accounts and bots stoking conflict by posting both pro- and anti-vaccine content^36^. During COVID-19, similar dynamics resurfaced globally, with state and non-state actors leveraging vaccine discourse to undermine confidence in public health authorities and democratic institutions^37–39^. Global observational work further links exposure to online disinformation with lower vaccination rates and diminished safety perceptions^40^.

Importantly, the spread of vaccine mis- and disinformation both reflects and intensifies political polarization, contributing to the erosion of institutional trust. Empirical research shows that susceptibility to misinformation is shaped by partisan identity and motivated reasoning, with individuals more likely to accept claims that align with their preexisting ideological commitments^23,41^. In vaccine-related discourse, misleading narratives often resonate because they tap into broader ideological frames—such as concerns about government overreach, pharmaceutical profiteering, or distrust of scientific elites^36,40^. Repeated exposure to such politicized misinformation has been linked to declining confidence in public health institutions and lower vaccination intentions^33,40^. Reviews of U.S. COVID-19 vaccine hesitancy further indicate that polarization and institutional distrust interact with misinformation exposure to reduce receptivity to corrective information and complicate the effectiveness of public health messaging^42^.

### Political polarization and shifting vaccine policy

Responses to vaccine programs and communication are profoundly shaped by political identity and polarization. While vaccine attitudes have always varied by social context, recent years have seen vaccination become a distinctly politicized issue in the United States, particularly as policy decisions and public discourse around vaccination have been closely aligned with partisan identity and governance priorities. Cross-national and U.S. evidence indicates that partisanship predicts vaccine confidence and uptake, with social networks composed of co-partisans and unvaccinated peers reinforcing hesitancy^43,44^. Studies of discussion networks further demonstrate polarization in who talks about vaccines with whom and how partisan identities map onto vaccination attitudes^45,46^.

During the COVID-19 pandemic, elite cues and mass media framing amplified these divides^47,48^. Public health recommendations—for masking, closures, and vaccination—were interpreted not merely as biomedical guidance but as signals of ideological stance, individual liberty, or government overreach^49,50^. As a result, in some contexts, vaccination decisions became expressive acts signaling political identity rather than purely health-related choices. Reviews of U.S. data conclude that polarization and institutional distrust interact with other determinants, such as perceived risk and safety concerns, to dampen responsiveness to public health messaging and complicate efforts to reach hesitant groups^42^.

In the post-pandemic period, vaccine policy changes under the Trump administration, directed by Health and Human Services Secretary Robert F. Kennedy Jr., have further intensified these dynamics. Federal actions have included revisions to the CDC childhood immunization schedule that reclassified certain vaccines from universal recommendations to categories emphasizing shared clinical decision-making or risk-based administration; restructuring of the Advisory Committee on Immunization Practices (ACIP), including replacement of prior members; and modifications to how vaccine safety monitoring and adverse event reporting are framed and communicated publicly. Supporters have characterized these changes as efforts to enhance transparency, parental choice, and regulatory efficiency. Critics—including professional medical associations, public health experts, and some state health departments—have argued that such changes risk weakening longstanding scientific advisory processes and may blur the boundary between political leadership and independent public health expertise. The American Academy of Pediatrics warned that removing universal recommendations for several routine childhood vaccines could be “dangerous and unnecessary” and undermine decades of evidence-based immunization guidance^51^.

### Institutional Distrust

A key downstream consequence of pervasive mis- and disinformation and intensifying political polarization is the erosion of institutional trust in government, scientific authorities, and public health agencies. Trust in institutions is widely recognized as a central determinant of vaccine confidence and uptake^7^. When public health guidance is perceived as inconsistent, politicized, or influenced by competing interests, individuals may question the credibility of both the message and the institutions delivering it. In highly polarized environments, institutional trust increasingly functions as a heuristic through which individuals evaluate health information, shaping whether recommendations from public health agencies are accepted, discounted, or interpreted through partisan lenses^52^.

Recent surveys suggest that institutional trust in the United States remains fragile, particularly in relation to government and public health authorities. National polling consistently finds that only a small minority of Americans report trusting the federal government to do what is right most of the time^53^. Although overall confidence in scientists remains relatively high compared with many other institutions, trust has become increasingly polarized along partisan lines since the COVID-19 pandemic^54^. Global surveys similarly document declining trust across major institutions—including government, media, and public health authorities—while highlighting misinformation and political polarization as key drivers of distrust^55^.

Declining institutional trust poses several distinct challenges for vaccine communication. First, it undermines the credibility of authoritative messengers. Messages delivered by federal agencies such as the Centers for Disease Control and Prevention or the Food and Drug Administration may be discounted among audiences who perceive these institutions as politically influenced. Second, distrust can increase reliance on alternative information sources—including partisan media outlets, social networks, or online influencers—that may circulate unverified or misleading claims^56^. Finally, distrust complicates corrective communication efforts, as fact-checks or evidence-based clarifications may be interpreted as attempts to defend institutional interests rather than neutral efforts to provide accurate information.

Importantly, declining trust can create a self-reinforcing cycle in which skepticism toward institutions increases receptivity to misinformation, and misinformation further erodes confidence in institutional authority. This dynamic makes vaccine communication particularly challenging, as the effectiveness of vaccine messages depends not only on message clarity but also on the perceived legitimacy of the institutions that produce it.

## Communication Strategies to Address Emerging Challenges

Navigating the challenges of misinformation and disinformation, political polarization and shifting vaccine policy, and institutional distrust requires moving beyond traditional deficit-based communication models that frame vaccine hesitancy as a simple knowledge gap. In contemporary media ecosystems, resistance to vaccination is often driven less by lack of information than by motivated reasoning, where individuals favor information consistent with preexisting beliefs, identity protection and group loyalty, and skepticism toward institutions. Effective communication strategies must therefore be anticipatory rather than reactive, relational rather than purely informational, and attentive not only to message content but also to identity dynamics, institutional credibility, and policy context.

### Proactive Inoculation and Prebunking

Because misinformation and disinformation now circulate as a persistent structural feature of digital environments, reactive myth correction is often insufficient and, in some cases, counterproductive. Inoculation theory offers a proactive alternative. Experimental research demonstrates that exposing individuals to weakened forms of misleading arguments—paired with refutations and explanations of common manipulation techniques—can build cognitive resistance to subsequent misinformation^57^. Rather than repeating specific falsehoods, prebunking interventions focus on forewarning audiences about rhetorical strategies such as cherry-picking, false expertise, conspiracy framing, or emotionally manipulative narratives.

Importantly, inoculation operates by strengthening individuals’ ability to recognize and reject misinformation independently, reducing reliance on constant corrective messaging. Systematic reviews indicate that inoculation-based interventions can meaningfully reduce susceptibility to vaccine misinformation, particularly when deployed prior to exposure^58^. In highly polarized contexts, where corrections may be interpreted as partisan rebuttals, prebunking approaches may be especially valuable because they emphasize critical reasoning skills rather than direct confrontation with identity-linked claims. As misinformation adapts rapidly to new policy debates and advisory changes, proactive resistance-building strategies may be more sustainable than case-by-case rebuttal efforts.

### Identity-congruent and community-based messengers

In politically divided environments, who delivers the message can matter as much as—or more than—what the message contains. Vaccine attitudes increasingly function as signals of political and social identity, meaning that communication perceived as originating from out-group individuals or institutions may be discounted regardless of evidentiary strength. Extensive evidence demonstrates that healthcare providers remain among the most trusted sources of vaccine information, and strong, presumptive recommendations from clinicians significantly increase uptake^22,59^. However, when federal agencies are perceived as politically influenced—particularly amid advisory restructuring or policy shifts—trust asymmetries can widen.

Under such conditions, identity-congruent messengers become central. Studies show that politically or religiously aligned messengers can be more persuasive in vaccine communication because they activate shared identity frameworks^60,61^. Community-based participatory approaches that engage faith leaders, local clinicians, educators, and civic organizations have demonstrated improved acceptability and, in some cases, uptake among marginalized and historically distrustful populations^24,62^. These strategies operate not merely by disseminating information but by leveraging relational trust embedded in existing social networks. When vaccination guidance is delivered by trusted in-group figures rather than distant institutions, it may circumvent identity-based filtering and reduce perceptions of political imposition.

### Cross-partisan appeals and value framing

Cross-partisan appeals seek to reposition vaccination within shared moral or civic values that transcend ideological divides. Rather than emphasizing partisan or technocratic arguments, these approaches highlight broadly endorsed goals such as protecting vulnerable populations, safeguarding community health, and maintaining social and economic stability. Research on vaccine communication shows that framing vaccination as a form of collective responsibility—emphasizing protection of children, older adults, or immunocompromised individuals—can increase vaccine acceptance and intentions across diverse populations^13,14^. Recent studies show the promise of using a naturalness appeal (vaccination as an immune-trainer) in vaccine communication^63^.

More broadly, communication and political psychology research suggests that value-congruent framing—aligning messages with audiences’ underlying moral commitments or values—can enhance message receptivity. Drawing on moral foundations theory, studies indicate that individuals respond more positively to persuasive appeals that resonate with their ideological value frameworks, such as appeals emphasizing care and harm prevention among liberal audiences or messages emphasizing duty, loyalty, and protection among conservative audiences^64^. Experimental work in political communication further shows that reframing contested issues using ideologically compatible moral language can reduce defensive processing and increase openness to opposing viewpoints^65^.

### Transparent policy communication

Shifting vaccine policies present a distinct communication challenge. Revisions to immunization schedules, restructuring of advisory committees, or changes in recommendation categories—regardless of intent—can be interpreted as evidence that prior guidance was unstable or politically influenced. In polarized contexts, policy change may amplify narratives that scientific recommendations shift with political leadership, thereby eroding institutional trust.

Effective communication during policy transitions requires proactive clarification of process and continuity. Research in risk communication suggests that transparency about decision-making criteria and acknowledgment of uncertainty can strengthen long-term trust, even when recommendations evolve^25,66^. Communicators should clearly distinguish between routine scientific updating and politically driven reinterpretation, explaining what has changed, what has not changed, and the evidentiary basis for revisions.

### Conclusion

Together, these strategies—proactive inoculation, identity-congruent messengers, cross-partisan appeals and value framing, and transparent policy communication—reflect a shift from message-centric persuasion toward trust-centric and identity-aware communication. In contemporary vaccine environments marked by misinformation, polarization, and declining institutional trust, strengthening cognitive resilience, relational trust, value alignment, and transparency may be more effective than relying solely on evidence transmission. Effective vaccine communication must therefore operate simultaneously at the levels of information, identity, and governance.

Importantly, communication resilience cannot depend exclusively on federal leadership. When governmental actors depart from established best practices in vaccine communication, the scientific community, professional medical organizations, state and local health departments, and civil society institutions assume a stabilizing role. Professional associations (e.g., pediatric, infectious disease, and public health societies) can issue independent evidence summaries and consensus statements that clarify the state of the science. Academic institutions and nonpartisan research bodies can enhance transparency by explaining advisory processes, evidentiary standards, and safety monitoring systems in accessible formats. State and local health agencies may coordinate messaging that reinforces continuity in clinical recommendations even amid federal shifts. Community leaders, clinicians, and trusted civic organizations can serve as intermediary communicators who contextualize policy developments for local audiences.

Citizens and individuals also play an important role in strengthening vaccine communication resilience. By critically evaluating information sources, verifying claims before sharing them, and relying on credible scientific and medical authorities, individuals can help slow the spread of misinformation within their social networks. Individuals can also contribute through respectful dialogue with peers, family members, and community members, particularly in contexts where institutional trust is limited. Because health decisions are often shaped within trusted interpersonal networks, informed citizens who model evidence-based reasoning and amplify credible information can help counter misinformation, bridge ideological divides, and reinforce social norms that support vaccination as a shared public good.

## Emerging Frontiers and Innovative Tools

Addressing the emerging challenges in vaccine communication requires not only refined communication strategies but also new tools capable of responding to rapidly evolving information environments. Traditional public health messaging alone may struggle to keep pace with the speed and scale of digital information ecosystems. As a result, scholars and practitioners are increasingly exploring innovative approaches that combine technological advances with behavioral insights and collaborative governance. Emerging efforts include social listening systems to monitor misinformation trends, artificial intelligence to detect and respond to misleading narratives, behavioral nudges to shape health decisions, and multi-stakeholder partnerships that coordinate responses across public health agencies, technology platforms, and community organizations.

### Social listening and real-time misinformation detection

Social listening—the systematic monitoring and analysis of online discussions—has become an essential tool for identifying emerging rumors, misinformation, and shifts in public sentiment around vaccines. During the COVID-19 pandemic, policy makers and public health professionals emphasized the value of deploying real-time infodemic monitoring systems to track trending narratives, identify misinformation “hot spots,” and guide rapid communication responses^67^. These systems combine automated data scraping from social media with human analysis to detect not only misinformation but also genuine concerns, questions, and informational gaps.

A growing body of research has evaluated the feasibility and effectiveness of social listening approaches. Karafillakis et al.^68^, in a scoping review, found that despite methodological challenges, social listening tools can successfully inform targeted risk communication strategies. A global study of social media conversations reported that negative tweets expressing vaccine skepticism, misinformation, or rumors tended to have larger audiences and generated greater engagement than positive or neutral content, highlighting the need for rapid responses to counter their influence and preserve public trust^69^. Complementary analyses of Twitter data further demonstrated that vaccine misinformation often circulates within polarized networks, reinforcing echo chambers and making continuous monitoring essential for identifying and reaching hesitant populations^70^.

Beyond isolated research efforts, social listening is increasingly being institutionalized within public health infrastructures. The World Health Organization (WHO) has developed standardized taxonomies and methodologies to generate integrated infodemic insights^71^, alongside digital platforms such as the WHO Early AI-supported Response with Social Listening (EARS) tool, which is designed to enhance monitoring at global, regional, and national levels^72^. These systems enable health authorities to identify prevailing questions, concerns, and information voids, as well as track emerging narratives and circulating mis- and disinformation within specific population groups. Regional initiatives are also taking shape: for instance, the Africa Infodemic Response Alliance (AIRA), launched in 2020, brings together international organizations and local partners to coordinate monitoring efforts and support national ministries of health in addressing vaccine misinformation^73^. Together, these initiatives illustrate how social listening is moving from an ad hoc practice to an integral component of immunization and risk communication strategies.

### Artificial Intelligence (AI) and Large Language Models (LLMs)

Advances in AI have opened new possibilities for vaccine communication. Early findings indicate that conversational AI can serve as a valuable supplement to human communication in vaccine promotion^74^. Recent studies show that AI-driven chatbots are capable of delivering accurate and empathetic responses to common vaccine questions, improving user satisfaction and, in some cases, increasing intention to vaccinate^75,76^. Pilot interventions further suggest that chatbots embedded within websites or designed for specific health contexts can extend reach to younger audiences and effectively address concerns tied to particular vaccines^77,78^.

Recent advances in the development of large language models (LLMs) have generated growing interest in their potential to enhance vaccine communication and health messaging in general. Unlike earlier rule-based or narrowly trained chatbots, LLMs offer greater versatility in producing context-specific, audience-tailored, and persuasive messages in real time. Karinshak et al.^79^ found that curated GPT-3–generated pro-vaccination messages were evaluated as more effective, presented stronger arguments, and elicited more positive attitudes than comparable human-authored messages released by the U.S. Centers for Disease Control and Prevention (CDC). More broadly, experimental evidence indicates that exposure to LLM-generated persuasive messages can significantly shift attitudes across a variety of policy issues compared to neutral control messages, underscoring their broader potential as communication tools^80^.

At the same time, critical challenges remain. AI tools risk amplifying misinformation if not carefully trained, and biases in training datasets can inadvertently reinforce inequities in communication. Privacy and data security are also pressing concerns, particularly when AI systems interact directly with users to collect personal information. Current consensus emphasizes that AI and LLMs should augment rather than replace human communicators, serving as scalable supports that free up human expertise for nuanced, context-specific engagement. If integrated responsibly, these tools could significantly enhance the reach, speed, and adaptability of vaccine communication in the face of evolving challenges.

### Behavioral economics and nudges

Nudging strategies—such as reminders, default appointments, or ownership framing—have shown consistent, measurable effects on vaccine uptake. In two large-scale randomized field experiments in the United States, Dai et al.^81^ demonstrated that simple text reminders and ownership-framed messages (e.g., “your dose is waiting for you”) significantly increased COVID-19 vaccination rates. Similarly, a mega-study involving more than 680,000 individuals confirmed that behavioral nudges, while modest in effect, reliably boosted influenza vaccination uptake across diverse populations^82^. These findings illustrate the practical value of low-cost, scalable interventions grounded in behavioral science for improving vaccination coverage.

### Cross-sector partnerships

Effective vaccine communication increasingly depends on collaboration between public health agencies, technology companies, community organizations, and non-governmental organizations. Analyses of COVID-19 response strategies show that partnerships between social media platforms and public health institutions—such as Facebook’s vaccine information hubs and YouTube’s misinformation policies—helped amplify credible content, although evaluations reveal mixed effectiveness and highlight the need for transparency and accountability^83,84^. Cross-sector collaboration can also include local NGOs co-designing campaigns with health departments to tailor messages for specific cultural or linguistic communities.

### Conclusion

Taken together, these emerging tools illustrate a shift toward more agile, participatory, and interdisciplinary approaches to vaccine communication. Social listening platforms can provide real-time situational awareness of emerging concerns and misinformation trends; artificial intelligence and large language models may enable scalable personalization of health messaging; behavioral nudges can leverage behavioral insights to produce modest but measurable improvements in vaccine uptake; and cross-sector partnerships can expand the reach and cultural relevance of communication efforts. Importantly, these approaches should be viewed as complementary tools that can enhance core communication strategies rather than as guaranteed solutions for increasing vaccination uptake. Their effectiveness depends on how they are integrated with mis- and disinformation mitigation, cross-partisan appeals and value framing, trusted messengers, transparent policy communication, and broader efforts to build institutional credibility. Moreover, their use raises important considerations regarding ethics, equity, and public trust. The future of vaccine communication will therefore depend on balancing technological and behavioral innovation with responsible implementation, ensuring that these tools strengthen—rather than undermine—the resilience of immunization systems.

## Research Gaps and Future Directions

Despite substantial progress in understanding vaccine communication, important gaps remain in how communication strategies are designed, evaluated, and adapted to contemporary information environments. A first priority is to address an important conceptual gap in the current literature concerning the distinction between vaccine hesitancy and vaccine refusal. While widely used, the SAGE definition of vaccine hesitancy—encompassing both delay and refusal—may obscure meaningful differences between individuals who are uncertain or ambivalent and those who hold firm convictions against vaccination. Distinguishing between these groups is critical, as they likely differ in underlying motivations, responsiveness to communication, and appropriate intervention strategies.

A second research priority is understanding vaccine communication within increasingly polarized and fragmented information ecosystems. While a growing body of work documents the influence of misinformation and partisan identity on vaccine attitudes, fewer studies have systematically tested strategies designed to counter these dynamics. Future research should examine how prebunking interventions, identity-congruent messengers, value-based framing, and community-based communication approaches perform under conditions of political polarization and institutional distrust. Experimental and field studies that examine how audiences interpret messages differently across ideological or media environments would help clarify which strategies can effectively bridge partisan divides.

Third, there is a need to improve the longitudinal evaluation of communication interventions. Much of the existing literature relies on short-term outcomes such as immediate changes in attitudes or vaccination intentions. However, vaccine confidence and behavior are dynamic and shaped by cumulative experiences over time. Few studies track whether initial communication effects persist, whether they translate into completed vaccination series, or how exposure to misinformation, policy changes, or evolving scientific guidance alters attitudes months or years later^12,58^. Longitudinal and panel-based studies could provide deeper insight into how trust, risk perceptions, and social norms evolve in response to communication efforts.

Another key gap involves the need for research that reflects greater geographic, cultural, and socioeconomic diversity. Much of the empirical evidence on vaccine communication strategies comes from high-income countries, particularly the United States and Western Europe. Expanding research to low- and middle-income countries, displaced populations, and communities historically marginalized by healthcare systems is essential for understanding how communication strategies perform across varied institutional contexts and cultural settings^62^. Comparative and cross-national studies can help identify which approaches are broadly applicable and which require substantial cultural adaptation.

Furthermore, greater attention is needed to the development and evaluation of emerging technological tools for vaccine communication. Social listening systems, artificial intelligence, and large language models offer new opportunities to monitor misinformation, generate tailored messages, and respond rapidly to evolving public concerns. Yet empirical evidence on their effectiveness, ethical implications, and long-term impact remains limited. Future studies should assess how these tools influence message credibility, public trust, and behavioral outcomes, while also examining risks such as algorithmic bias, privacy concerns, and the potential for AI systems to inadvertently amplify misinformation.

Finally, vaccine communication research must increasingly be integrated with broader efforts to strengthen immunization system resilience. The COVID-19 pandemic illustrated that communication cannot be treated as a peripheral component of vaccination programs. Instead, it must be embedded across planning, rollout, monitoring, and crisis response. Building durable communication infrastructures—such as real-time rumor monitoring systems, trusted community advisory networks, and coordinated partnerships between public health agencies, technology platforms, and civil society—can enable faster and more adaptive responses to emerging threats^66,67^. Advancing this agenda will require interdisciplinary collaboration among communication scholars, epidemiologists, behavioral scientists, data scientists, and community stakeholders.

## Conclusion

Vaccine communication stands at a critical juncture. Decades of research confirm that tailored, evidence-based strategies, when implemented thoughtfully, can bolster vaccine confidence and uptake. However, the contemporary communication environment presents challenges that extend beyond traditional message design. The rapid spread of misinformation and disinformation, intensifying political polarization, shifting vaccine policy debates, and declining institutional trust have created conditions in which even well-designed communication efforts may struggle to achieve their intended effects. These developments highlight that effective vaccine communication must address the broader social, political, and technological systems that shape how health information is interpreted and acted upon.

At the same time, emerging tools and approaches offer new opportunities to strengthen vaccine communication. Innovations such as social listening systems, artificial intelligence–enabled messaging, behavioral nudges, and cross-sector partnerships can help monitor public concerns, respond more rapidly to misinformation, and tailor communication to diverse audiences. Yet technological innovation cannot substitute for the foundational elements of trust, transparency, and community engagement that sustain public confidence in vaccination.

Moving forward, advancing the field will require sustained interdisciplinary and cross-cultural research to understand how communication strategies function in polarized and digitally mediated environments. Longitudinal studies are particularly needed to assess whether interventions produce durable effects on vaccine confidence and behavior. Integrating communication expertise into the design and governance of immunization systems will also be essential. By balancing innovation with responsibility and grounding communication in both evidence and community partnership, vaccine communication can strengthen the resilience of immunization systems in an increasingly complex informational landscape.

## Data Availability

No datasets were generated or analysed during the current study.
